# The long-term effect of social, educational, and health expenditures on indicators of life expectancy: an empirical analysis for the OECD countries

**DOI:** 10.3389/fpubh.2024.1497794

**Published:** 2024-12-18

**Authors:** Levent Aytemiz, Gamze Sart, Yilmaz Bayar, Marina Danilina, Funda H. Sezgin

**Affiliations:** ^1^Department of Public Finance, Faculty of Economics and Administrative Sciences, Bandirma Onyedi Eylul University, Bandirma-Balikesir, Türkiye; ^2^Department of Educational Sciences, Hasan Ali Yucel Faculty of Education, İstanbul University-Cerrahpaşa, İstanbul, Türkiye; ^3^Department of Economics, Plekhanov Russian University of Economics (PRUE), Moscow, Russia; ^4^Department of Economics, Financial University Under the Government of the Russian Federation, Moscow, Russia; ^5^Department of Industrial Engineering, Istanbul University-Cerrahpaşa, İstanbul, Türkiye

**Keywords:** public social expenditures, public education expenditures, health expenditures, indicators of life expectancy, OECD states, panel cointegration analysis

## Abstract

Life expectancy provides insights into population health and the socio-economic development level of a country. However, there has been a significant gap in life expectancy between developed and underdeveloped countries, although these countries and international institutions have focused on reducing these inequalities. This article explores the long-term effects of social, educational, and health expenditures together with GDP *per capita* on indicators of life expectancy in the OECD states over the period of 2005–2021 through second-generation cointegration analysis. The results of the cointegration analysis demonstrate that public social and educational expenditures, health expenditures, and real GDP *per capita* have a positive effect on indicators of life expectancy in the OECD states over the long term, but the effect of real GDP *per capita* and health expenditures on indicators of life expectancy is relatively higher than effect of public social and educational expenditures on indicators of life expectancy. In conclusion, the effective use of governmental resources in terms of social, educational, and health expenditures can be beneficial in improving population health directly and indirectly through economic growth and development.

## 1 Introduction

Life expectancy indicates the average lifespan of a society and increases in life expectancy generally reflect progress in living standards, medicine, education, and lifestyle (1). Therefore, life expectancy is generally accepted as one of the key indicators of a country’s socioeconomic development (2). On the other hand, healthy human capital is one of the main factors behind economic growth and development (3). In conclusion, every country tries to improve life expectancy by implementing economic, social, educational, and health policies that address the aforementioned implications for society.

All parts of the world have experienced improvements in life expectancy, and inter-country differences in life expectancy have also decreased during the period of 1990–2022. In this context, Chad (52.997 years), Lesotho (53.036 years), and Nigeria (53.633 years) had the lowest life expectancy at birth (LEB) in 2022, while Japan (84.82 years), Liechtenstein (84.656 years), and Switzerland (84.255 years) recorded the highest LEB in 2022 (4). The gap between the highest and the lowest LEB of the countries (Japan and South Sudan) in 1990 was 49.049 years, but this gap decreased to 31.823 years in 2022 between Japan and Chad (4). Although there has been a partial improvement in the life expectancy gap between countries, the gap remains significant. This underlines the importance of emphasizing the Sustainable Development Goals (SDGs) outlined in the 2030 Agenda for Sustainable Development (5). Aksan and Chakraborty (6) also noted a convergence in LEB, but a divergence in life expectancy at age 65 between 1960 and 2015 worldwide.

Researchers have also conducted a wide range of empirical studies on economic, social, and environmental factors behind inter-country differences in life expectancy. The factors listed in Table 1 have been identified as the major drivers of various life expectancy indicators. In this context, researchers have primarily focused on the impact of factors related to national and personal income, health expenditures, and infrastructure on life expectancy. However, the effects of other social, economic, environmental, and demographic factors, such as institutional quality, financial sector development, government effectiveness, poverty, income inequality, lifestyle, demographic indicators, the environment, information and communication technologies, unemployment and employment, education, population, urbanization, and political regimes on life expectancy have also been explored, albeit to a relatively lesser extent.

**Table 1:** Economic, institutional, social, and environmental drivers of life expectancy.

Drivers of life expectancy	Empirical studies
National income, income *per capita*	Tarcă et al. (2), Lin et al. (38), Ali and Ahmad (39), Monsef and Mehrjardi (40), OECD (41), Aydin (42), Păunică et al. (43), Wirayuda and Chan (44), Aanegola et al. (45), Karma (46), Anwar et al. (47), Roffia et al. (8)
Income inequality	Wirayuda and Chan (44), Ahmad et al. (56)
Poverty	Aanegola et al. (45)
Health expenditures, health infrastructure, health care system	Tarcă et al. (2), OECD (41), Aydin (42), Wirayuda and Chan (44), Aanegola et al. (45), Anwar et al. (47), Roffia et al. (8), Ahmad et al. (56), Sango-Coker and Bein (57), Gedikli et al. (58), Khan et al. (59)
Social expenditures	Roffia et al. (8), van den Heuvel and Olaroiu (16), Reynolds and Avendano (9)
Lifestyle	OECD (41), Welsh et al. (60), Wirayuda and Chan (44)
Government effectiveness	Khan et al. (59)
Food production, final consumption, nutritional status	Lin et al. (38), Ali and Ahmad (39), Wirayuda and Chan (44)
Education	Lin et al. (38), Ali and Ahmad (39), OECD (41), Wirayuda and Chan (44), Karma (46)
Unemployment and employment	Monsef and Mehrjardi (40), Aydin (42), Wirayuda and Chan (44), Roffia et al. (8)
Inflation	Monsef and Mehrjardi (40), Wirayuda and Chan (44)
Population size	Ali and Ahmad (39), Wirayuda and Chan (44), Roffia et al. (8)
Marital status	Karma (46)
Gender inequality	Wirayuda and Chan (44)
Infant mortality rate	Wirayuda and Chan (44)
Fertility rate	Karma (46)
Chronic respiratory diseases	Roffia et al. (8)
Environment	Tarcă et al. (2), Ali and Ahmad (39), Wirayuda and Chan (44), Karma (46), Anwar et al. (47)
Urbanization	Karma (46), Ahmad et al. (56)
Political regime	Lin et al. (2)
Information and communication technologies	Wang et al. (61), Bayar et al. (49)
Financial development, financial inclusion	Nica et al. (50), Banerjee et al. (62)
Institutional quality	Nica et al. (50)

This research surveys the long-term effects of social, educational, and health expenditures together with real GDP *per capita* on indicators of life expectancy, as these variables have the ability to impact life expectancy through multiple channels. Social expenditures include cash benefits, delivery of goods and services, and tax reductions for low-income households, the unemployed, the disabled, the sick, the older adults, or young individuals (7). Therefore, social expenditures can positively impact life expectancy by increasing the disposable income or consumption levels of individuals (8). Furthermore, increases in household disposable income can also improve life expectancy through higher living standards, better education opportunities, improved medical care, healthier lifestyles, and reduced stress levels (8, 9).

Public education investments can also impact life expectancy by improving education, which enables individuals to attain better living standards, healthy lifestyles, and healthcare opportunities. This effect arises because individuals with higher education levels generally earn higher wages (10, 11). On the other hand, increases in health expenditures can positively affect the life expectancy through improvements in health infrastructure and care, healthy nutrition and immunization (12). Lastly, social, educational, and health expenditures can foster economic growth and development via increased productivity mainly resulting from improvements in human capital and, in turn, improve life expectancy through enhancements in income and health facilities (11–14).

A person with relatively higher income is expected to live longer due to easier access to healthy life style and appropriate healthcare. Preston (15) also suggested that the persons in higher-income countries generally have higher lifetime than persons in the lower-income countries. However, increases in income cause higher increases in lifetime at low GDP *per capita* values, but relatively lower increases in lifetime emerge at higher GDP *per capita* values (15). In conclusion, a positive association between real GDP *per capita* and indicators of life expectancy is anticipated.

This research investigates the long-term effects of social, educational, and health expenditures and real GDP *per capita* on indicators of life expectancy in OECD countries, because OECD includes countries with different economic, social, and institutional characteristics. This article intends to contribute to the associated empirical literature from three perspectives. First, the empirical studies have usually investigated the effect of various economic, social, and institutional factors on life expectancy at birth. Our article is also utilized healthy life expectancy at birth and 60 and life expectancy at 60 together with life expectancy at birth. Secondly, in the associated empirical literature, only Roffia et al. (8), van den Heuvel and Olaroiu (16), Reynolds and Avendano (9), Dutton et al. (17), Aydan et al. (18), Cardona et al. (19), and Aydan et al. (20) have been explored the effect of social expenditures on life expectancy in the short term. But this research investigates the long-term effects of social expenditures on indicators of life expectancy at birth and 60 differently from the empirical literature by means of second-generation cointegration test which enables us to determine whether the variables under consideration have a stable long-run relationship (21). Thirdly, this research examines the effect of public educational investment on indicators of life expectancy distinguishing it from the literature on the nexus between education indicators and population health. In the remainder of the article, the associated empirical literature is reviewed in Section 2, data, data sources, and econometric tests are explained in Section 3. Econometric tests and discussions about the empirical findings are presented in Section 4, while the conclusions and policy suggestions are outlined in Section 5.

## 2 Literature review

Life expectancy is one of the essential proxies for population health and socio-economic development. The gap in life expectancy at the global level has partially narrowed in recent years, but a significant gap between developed and underdeveloped countries persists (4). Therefore, economic, environmental, social, and health-related factors contributing to the heterogeneity in life expectancy among countries have been widely explored. In this research, the long-term effects of social, educational, and health expenditures on life expectancy in OECD states have been investigated because only a few academicians have empirically studied the short-term effects of these expenditures. Furthermore, the impacts of governmental social and educational expenditures on life expectancy are more discernible over the long term.

Empirical studies analyzing the interplay between social expenditures and life expectancy across various countries and panels have generally utilized the regression approach, revealing an increasing effect of both public and private social spending on life expectancy in compatible with the theoretical considerations (8, 9, 16–20).

Roffia et al. (8) investigated the effects of health-related indicators, as well as social, economic, and environmental factors, on life expectancy at birth in OECD countries between 1999 and 2018 using a regression approach. They identified a positive effect of social expenditures on life expectancy. On the other hand, van den Heuvel and Olaroiu (16) explored the effects of health-related indicators, education, and social protection expenditures on life expectancy in 31 European countries using a regression approach and found a positive effect of social protection expenditures on life expectancy.

Reynolds and Avendano (9) investigated the influence of socio-economic factors on life expectancy in 20 high-income countries from 1980 to 2010 using a regression approach and discovered a positive influence of incapacity benefits on life expectancy. Dutton et al. (17) analyzed the effect of provincial social expenditures on life expectancy in Canada from 1981 to 2011 using a regression approach and unveiled a positive influence of social spending on life expectancy.

Aydan et al. (18) explored the effect of health and social expenditures on life expectancy among OECD members from 2006 to 2017 using regression analysis and disclosed an increasing influence of social expenditures on life expectancy. Cardona et al. (19) investigated the nexus between spending on social services and life expectancy in the United States from 2005 to 2010 using structural equation modeling and revealed a positive effect of social expenditures on life expectancy. Lastly, Aydan et al. (20) analyzed the effect of social expenditures, economic freedom, and health expenditures on well-being proxied by better life index among OECD members from 2013 to 2017 using regression analysis and discovered a positive influence of social expenditures on well-being.

The interaction between educational indicators, including literacy rate, schooling years, and education index of United Nations Development Program, and life expectancy has been studied in relatively more research papers, as summarized in Table 2. These research papers typically utilized a panel-level regression approach and uncovered a positive influence of various education indicators on life expectancy (11, 22–34). However, Hazan (35) suggested that the influence of education on life expectancy can vary based on the proxies of life expectancy used. Bilas et al. (36) disclosed a decreasing influence of education on life expectancy in European countries. Last, Craigwell et al. (37) unveiled an insignificant effect of public education spendings on primary and secondary school enrolments. This article investigates the effect of public education investments, proxied by public educational expenditures, on life expectancy, differing from most of the studies presented in Table 2.

**Table 2:** Literature summary on the interplay between life expectancy and education proxies.

Research article	Sample; duration	Methods	Results
Moga Rogoz et al. (11)	New EU members; 2000–2019	Cointegration and causality analyses	Positive
Yavari and Mehrnoosh (22)	89 countries; 1986	Regression	Positive
Kabir (23)	91 developing countries;	Regression	Positive
Bayati et al. (24)	21 East Mediterranean economies; 1995–2007	Regression	Positive
Delavari et al. (25)	Iran; 1985–2013	Cointegration and regression	Positive
Hassan et al. (26)	108 developing economies; 2006–2010	Regression	Positive
Ketenci and Murthy (27)	the United States; 1960–2012	Cointegration	Positive
Lutz and Kebede (28)	174 states; 1970–2015	Regression	Positive
Luy et al. (29)	Italy, Denmark, and the USA; 1990–2010	Replacement decomposition	A strong interaction between education and population health
Raghupathi and Raghupathi (30)	26 OECD states; 1995–2015	Correlation	Positive
Case and Deaton (31)	the United States; 1990–2018	Life table methods	Positive
Siegel et al. (32)	Germany; 2015–2017	Regression	Positive
Lleras-Muney et al. (33)	The United States;	Regression	Positive
Balaj et al. (34)	Web of Science, Scopus, PubMed, Embase, EconLit, Global Health (CAB), and Sociology; 1980–2023	Systematic review and meta-analysis	Each additional year of education caused an average decrease in mortality risk of 1.9%.
Hazan (35)	37 and 61 countries; 1960–1990	Correlation	Positive for life expectancy at birth, but insignificant for life expectancy at age 5
Bilas et al. (36)	EU states; 2001–2011	Regression	Negative
Craigwell et al. (37)	19 Caribbean countries; 1980–2009	Regression	Insignificant effect of public education spendings on primary and secondary school enrolments

The relationship between health expenditures and life expectancy have been widely investigated and the empirical studies in Table 3 have unveiled a positive effect of health expenditures on life expectancy. However, van den Heuvel and Olaroiu (16) found that healthcare expenditures are not one of the main drivers of life expectancy. Last, Tarcă et al. (2), Lin et al. (38), Ali and Ahmad (39), Monsef and Mehrjardi (40), OECD (41), Aydin (42), Păunică et al. (43), Wirayuda and Chan (44), Aanegola et al. (45), Karma (46), Anwar et al. (47), Roffia et al. (8) also suggested the income as one of the main drivers of life expectancy.

**Table 3:** Literature summary on the interplay between life expectancy and health expenditures.

Research article	Sample; duration	Methods	Results
Tarcă et al. (2)	13 Eastern European states; 2000–2020	Regression	Positive
OECD (41)	OECD countries; 1995–2015	Regression	Positive
Aydin (42)	OECD countries; 2000–2016	Regression	Positive
Wirayuda and Chan (44)	46 articles; 2004–2019	Literature review	Positive
Aanegola et al. (45)	183 countries; 2000–2016	Causal approach	A positive effect of private health expenditures
Anwar et al. (47)	OECD countries; 1996–2020	Regression	Positive
Roffia et al. (8)	OECD countries; 1999–2018	Regression	Positive
Ahmad et al. (56)	South Asian countries; 1997–2021	Regression	Positive
Sango-Coker and Bein (57)	African countries; 1999–2014	Regression	Positive
Gedikli et al. (58)	Turkey, Azerbaijan, Kazakhstan, Kyrgyzstan, Tajikistan, Turkmenistan and Uzbekistan; 2000–2015	Cointegration analysis	Positive
Khan et al. (59)	Pakistan; 2000–2020	Cointegration analysis	Positive
van den Heuvel and Olaroiu (16)	31 European countries	Regression	Insignificant relationship between health expenditures and life expectancy

Based on the explored literature, the research hypotheses of our research article are established as follows:

*HP*1: Public social expenditures have a positive effect on indicators of life expectancy in the long-term.

*HP*2: Public education expenditures have a positive effect on indicators of life expectancy in the long-term.

*HP*3: Health expenditures have a positive effect on indicators of life expectancy in the long-term.

*HP*4: Real GDP per capita has a positive effect on indicators of life expectancy in the long-term.

## 3 Data and methods

The article examines the long-term effects of social, educational, and health expenditures together with real GDP *per capita* on indicators of life expectancy using the Westerlund and Edgerton (48) bootstrap cointegration test. The variables considered in the econometric applications are listed in Table 4. Population health is proxied by healthy life expectancy at birth and 60, life expectancy at birth and 60 and these variables are sourced from World Health Organization (49, 50). Public social and educational expenditures are represented by total public social expenditures and government expenditure on education as a percentage of GDP, respectively. Public social expenditures are sourced from the OECD (51), and government education expenditures are obtained from the World Bank (36). Health expenditures are proxied by current health expenditure as a percent of GDP and income is represented by GDP *per capita* based on constant 2015 US$. Both variables are, respectively, provided from World Bank (52, 53). The variables of social, educational, and health expenditures are available as a percent of GDP in databases of OECD and World Bank. This form of data enables us to compare the variables among countries and/or over time in connection with the overall size of the economy (36).

**Table 4:** Description of variables.

Variable proxies	Data identification	Data sources
HLEB	Healthy life expectancy at birth (years)	WHO (52)
HLE60	Healthy life expectancy at 60 (years)	WHO (52)
LEB	Life expectancy at birth (years)	WHO (53)
LE60	Life expectancy at 60 (years)	WHO (53)
SOCEX	Public social expenditures (% of GDP)	OECD (7)
EDEX	Education expenditures (% of GDP)	World Bank (7)
HEX	Current health expenditure (% of GDP)	World Bank (63)
INCOME	GDP *per capita* (constant 2015 US$)	World Bank (64)

The sample of the research includes 32 OECD members (Austria, Belgium, Canada, Chile, Czechia, Denmark, Estonia, Finland, France, Germany, Greece, Hungary, Iceland, Ireland, Israel, Italy, Japan, Korea, Latvia, Lithuania, Luxembourg, Netherlands, Norway, Poland, Portugal, Slovak Republic, Slovenia, Spain, Sweden, Switzerland, the United Kingdom, and the United States), selected due to the availability of public social and educational expenditures. The dataset spans from 2005 to 2021, as government expenditure on education is available starting in 2005 for most OECD states and continues until 2021.

The econometric analyses are conducted using statistical packages Gauss 12.0, EViews 10.0, and Stata 17.0. According to the summary indicators in Table 5, the average of healthy life expectancy and life expectancy at birth are, respectively, 68.970 years and 78.863 years while the average of healthy life expectancy and life expectancy at 60 are 17.441 years and 22.491 years. On the other hand, the average values of total public social and educational expenditures and health expenditures as a percentage of GDP are 21.106 and 5.185%, and 8.654% of GDP, respectively. Lastly, the average of real GDP *per capita* is USD 37033.040 While real GDP *per capita* and public social expenditures show a remarkable variation among OECD states, indicators of life expectancy, public educational expenditures, and health expenditures have been relatively more stable during the period from 2005 to 2021.

**Table 5:** Summary indicators of the variables.

Characteristic	HLEB	HLE60	LEB	LE60	SOCEX	EDEX	HEX	INCOME
Mean	68.970	17.441	78.863	22.491	21.106	5.185	8.654	37033.040
Median	69.400	17.800	79.700	22.900	21.100	4.976	8.780	35669.790
Maximum	73.600	20.500	84.200	26.400	34.900	8.614	13.035	112417.900
Minimum	61.600	13.700	69.800	18.000	5.900	2.930	4.548	8554.694
Std. Dev.	2.193	1.391	2.970	1.795	5.550	1.134	1.798	22286.670
Skewness	−0.963	−0.734	−1.046	−0.558	−0.149	0.745	−0.103	1.207
Kurtosis	3.850	2.905	3.490	2.611	2.571	3.294	2.064	4.462

The correlation matxrix among the explanatory variables is displayed in Table 6. The correlation matrix shows us that a positive correlation among social expenditures, educational expenditures, health expenditures, and income proxied by real GDP *per capita*. Furthermore, relatively low correlation coefficients demonstrate the non-existence of multicollinearity problem.

**Table 6:** Correlation matrix.

	SOCEX	EDEX	HEX	INCOME
SOCEX	1.000	0.330**	0.313**	0.155**
EDEX		1.000	0.308**	0.286**
HEX			1.000	0.271**
INCOME			0.271**	1.000

**significant at 5%.

The effect of total public social and educational expenditures, health expenditures, and income on healthy life expectancy at birth and 60 and life expectancy at birth and 60 among OECD members is investigated using four models presented in Equations 1–4. The dependent variables are healthy life expectancy at birth (HLEB), healthy life expectancy at 60 (HLE60), life expectancy at birth (LEB) and life expectancy at 60 (LE60). On the other hand, the independent variables are public social expenditures (SOCEX), government expenditure on education (EDEX), health expenditures (HEXP), and real GDP *per capita* (INCOME).


(1) $$\begin{array}{l} HLEB_{\mathrm{it}}=\alpha_{i}+\beta_{1}\mathrm{SOCE}X_{\mathrm{it}}+\beta_{2}EDEX_{\mathrm{it}}+\beta_{3}\mathrm{HE}X_{\mathrm{it}}\\ +\beta_{4}\mathrm{INCOM}E_{\mathrm{it}}+\varepsilon_{\mathrm{it}} \end{array}$$

(2) $$\begin{array}{l} HLE60_{\mathrm{it}}=\alpha_{i}+\beta_{1}\mathrm{SOCE}X_{\mathrm{it}}+\beta_{2}EDEX_{\mathrm{it}}+\beta_{3}\mathrm{HE}X_{\mathrm{it}}\\ +\beta_{4}\mathrm{INCOM}E_{\mathrm{it}}+\varepsilon_{\mathrm{it}} \end{array}$$

(3) $$\begin{array}{l} LEB_{\mathrm{it}}=\alpha_{i}+\beta_{1}\mathrm{SOCE}X_{\mathrm{it}}+\beta_{2}EDEX_{\mathrm{it}}+\beta_{3}\mathrm{HE}X_{\mathrm{it}}\\ +\beta_{4}\mathrm{INCOM}E_{\mathrm{it}}+\varepsilon_{\mathrm{it}} \end{array}$$

(4) $$\begin{array}{l} LE60_{\mathrm{it}}=\alpha_{i}+\beta_{1}\mathrm{SOCE}X_{\mathrm{it}}+\beta_{2}EDEX_{\mathrm{it}}+\beta_{3}\mathrm{HE}X_{\mathrm{it}}\\ +\beta_{4}\mathrm{INCOM}E_{\mathrm{it}}+\varepsilon_{\mathrm{it}} \end{array}$$

where i and t demonstrate the OECD members and years, respectively.

In the econometric application of this article, cross-sectional dependency (CD) tests and homogeneity tests are initially performed to select appropriate cointegration and unit root tests. In this context, the stationarity of the series under consideration is analyzed using the Pesaran (54) CIPS test due to CD among the variables described in four models.

Subsequently, the cointegration relationship among the variables is examined using Westerlund and Edgerton’s (48) LM cointegration test to see the long-term relationship among social, educational, and health expenditures, real GDP *per capita*, and indicators of life expectancy. This test produces robust long-term coefficients in case of CD through bootstrapping (48) unlike the traditional cointegration tests. Otherwise, asymptotic approach is used. Furthermore, Westerlund and Edgerton’s (48) LM cointegration test allows for dependence both between and within the cross-sections in the cointegration equation. Last, the test is is proven to be efficient in small datasets (48). In conclusion, Westerlund and Edgerton’s (48) LM cointegration test is selected considering the presence of CD and our dataset size. The test is derived from Equations 5–7.


(5) $$y_{\mathrm{it}}=\alpha_{i}+x_{\mathrm{it}}\beta_{\mathrm{it}}+Z_{\mathrm{it}}$$

(6) $$Z_{\mathrm{it}}=\mu_{\mathrm{it}}+V_{\mathrm{it}}=\overset{t}{\underset{J=1}{\sum }}{ŋ}_{ij}$$

i and t demonstrate the OECD countries and the years of the panel. $Z_{\mathrm{it}}$ is the disturbance term. LM statistic of the cointegration test is figured out as following:


(7) $$LM_{N}^{+}=\frac{1}{NT^{2}}\overset{N}{\underset{i=1}{\sum }}\overset{T}{\underset{t=1}{\sum }}{\hat{w}}_{i}^{-2}s_{\mathrm{it}}^{2}$$

In Equation 7, $s_{\mathrm{it}}^{2}$ is partial total of $Z_{\mathrm{it}}$ and ${\hat{w}}_{i}^{-2}$ is long-term variance of $\mu_{\mathrm{it}}$. Both are derived from a cointegration model using fully modified ordinary least squares. The null hypothesis proposes the presence of a cointegration relationship among the variables under consideration. Asymptotic and bootstrap critical values are generated from normal distributions and bootstrapping, respectively, to test the hypotheses. Bootstrap *p* values are used if CD exists among the variables; otherwise, asymptotic p values are applied.

Finally, the AMG (Augmented Mean Group) estimator, introduced by Eberhardt and Bond (55), is employed to estimate the panel and cross-sections’ cointegration coefficients. This estimator accounts for CD and heterogeneity and provides coefficients for both the panel and individual OECD countries. The estimation by the AMG is implemented at two stages. At the first stage, model is estimated through the first differences of the variables as in Equation 8 because non-stationary variables and unobservable factors produce biased results in the regression with the level values of the variables. Thus, time dummy variables of ${\hat{\mu }}_{t}^{*}$ are obtained (55).


$$\Delta y_{\mathrm{it}}=b^{\prime }\Delta X_{\mathrm{it}}+\overset{T}{\underset{t=2}{\sum }}c_{t}\Delta D_{t}+e_{\mathrm{it}}$$

(8) $$\to {\hat{c}}_{t}\text{≡}{\hat{\mu }}_{t}^{*}$$

At the second stage, the model in Equation 9 is estimated. The time dummy variable is included in the regression of each cross-section. AMG estimations are calculated as the average of each cross-section coefficient (55).


$$y_{\mathrm{it}}=\alpha_{i}+b_{i}^{\prime }x_{\mathrm{it}}+c_{i}t+d_{i}{\hat{\mu }}_{t}^{*}+e_{\mathrm{it}}$$

(9) $${\hat{b}}_{AMG}=N^{-1}\underset{i}{\sum }{\hat{b}}_{i}$$

## 4 Results and discussion

In the application section of our research paper, cross-sectional dependency (CD) and heterogeneity are initially examined using CD and delta tilde tests, respectively. The CD among the series is analyzed using LM, LM CD, and LM_adj._ tests, and the results of these tests are presented in Table 7. The null hypothesis favoring CD independence is rejected because the *p*-values of the LM, LM CD, and LM_adj._ tests are below 5%. Consequently, the CD among the variables is confirmed.

**Table 7:** Results of LM, LM CD, and LM_adj._ tests.

CD tests	Model-1	Model-2	Model-3	Model-4
	Test statistics	Test statistics	Test statistics	Test statistics
LM	974.8***	983***	938.9***	1024***
LM CD	21.34***	21.39***	15.41***	19.56***
LM_adj._	21.01***	21.48***	18.96***	23.78***

***significant at 1%.

The presence of heterogeneity is also explored using delta tilde tests, and the findings are presented in Table 8. The null hypothesis of homogeneity for both delta and adjusted delta tests is rejected, confirming the presence of heterogeneity. Based on the results of the CD and heterogeneity tests, it is recommended that tests of cointegration and unit roots that consider CD and heterogeneity are preferred due to their relatively more robust outcomes.

**Table 8:** Consequences of delta tilde tests.

Homogeneity tests	Model-1	Model-2	Model-3	Model-4
	Test statistics	Test statistics	Test statistics	Test statistics
$\tilde{\Delta }$	14.414***	14.354***	13.304***	15.140***
$\tilde{\Delta }adj\text{.}$	17.920***	17.844***	16.539***	18.822***

***significant at 1%.

The presence of unit roots in the series employed in the cointegration analysis is examined using the panel CIPS test, due to the presence of cross-sectional dependency. The CIPS test statistics are presented in Table 9. The results of the unit root tests reveal that the series include unit roots at level values. However, the first differences of the variables do not exhibit unit roots and become stationary.

**Table 9:** Consequences of CIPS test.

Variable	Level		1. Level	
	Constant	Constant + Trend	Constant	Constant + Trend
HLEB	−0.932	−1.107	−6.907**	7.223**
HLE60	−0.815	−0.916	−8.221**	−8.769**
LEB	−0.974	−0.995	−7.866***	−8.104***
LE60	−1.043	−1.114	−9.092**	−9695**
SOCEX	−1.276	−1.288	−7.584***	−8.104***
EDEX	−1.061	−1.123	−8.303***	−8.995***
HEX	−1.290	−1.300	−9.764***	−10.137***
INCOME	−0.981	−1.088	−10.032***	−10.554***

*** and *** are, respectively, significant at 1 and 5%.

The cointegration relationship among indicators of life expectancy, social and educational spending, health expenditures and real GDP *per capita* is investigated using the Westerlund and Edgerton (48) LM cointegration test. The LM test statistics, along with bootstrap and asymptotic *p*-values, are presented in Table 10. Due to the bootstrap p-values and the presence of cross-sectional dependency, the null hypothesis, which posits the existence of a long-term relationship among the indicators of life expectancy, social, educational, and health expenditures, and real GDP *per capita*, is accepted.

**Table 10:** Consequences of Westerlund and Edgerton Cointegration test.

Model 1					
Constant			Constant and trend		
LM Statistic	Asymptotic *p* value	Bootstrap *p* value	LM Statistic	Asymptotic *p* value	Bootstrap *p* value
7.584	0.214	0.296	8.112	0.311	0.346
Model 2					
8.365	0.316	0.385	9.330	0.412	0.458
Model 3					
8.973	0.378	0.405	9.504	0.423	0.470
Model 4					
6.862	0.287	0.329	7.476	0.348	0.402

The AMG estimator is used to estimate the long-term cointegration coefficients at both panel and cross-sectional levels, and these coefficients are presented in Tables 11, 12. Table 11 demonstrates that social expenditures positively impact healthy life expectancy at birth and 60 in nearly all OECD countries except Estonia, Latvia, Lithuania, and Slovakia, but educational and health expenditures and real GDP *per capita* positively affect healthy life expectancy at birth and 60 in all OECD members. Impact of income proxied by real GDP *per capita* and health expenditures on healthy life expectancy at birth and 60 is found to be relatively much higher than impact of public social and educational expenditures on healthy life expectancy at birth and 60. Furthermore, the long-term coefficients indicate that impact of all variables on healthy life expectancy at birth are generally higher than the impact of all variables on healthy life expectancy at 60.

**Table 11:** Long-term coefficients (Model 1 and 2).

OECD Countries	Model 1				Model 2			
	SOCEX	EDEX	HEX	INCOME	SOCEX	EDEX	HEX	INCOME
Austria	0.124**	0.093**	0.213**	0.242**	0.117**	0.088**	0.208**	0.236**
Belgium	0.123**	0.081**	0.228**	0.231**	0.115**	0.074**	0.216**	0.225**
Canada	0.128**	0.067**	0.238**	0.246**	0.121**	0.062**	0.228**	0.237**
Chile	0.125**	0.090**	0.211**	0.239**	0.119**	0.089**	0.205**	0.228**
Czechia	0.154**	0.053**	0.182**	0.221**	0.134**	0.055**	0.174**	0.205**
Denmark	0.133**	0.085**	0.256**	0.263**	0.121**	0.072**	0.247**	0.251**
Estonia	0.122	0.092**	0.173**	0.185**	0.112	0.084**	0.161**	0.174**
Finland	0.128**	0.083**	0.254**	0.268**	0.123**	0.080**	0.243**	0.256**
France	0.119**	0.101**	0.259**	0.267**	0.105**	0.099**	0.248**	0.250**
Germany	0.120**	0.092**	0.255**	0.284**	0.117**	0.084**	0.242**	0.272**
Greece	0.123**	0.098**	0.234**	0.237**	0.116**	0.091**	0.227**	0.220**
Hungary	0.129**	0.087**	0.171**	0.189**	0.123**	0.079**	0.164**	0.173**
Ireland	0.213**	0.080**	0.185**	0.193**	0.178**	0.077**	0.176**	0.184**
Israel	0.218**	0.073**	0.164**	0.178**	0.197**	0.065**	0.158**	0.161**
Italy	0.117**	0.106**	0.177**	0.183**	0.109**	0.093**	0.168**	0.179**
Japan	0.124**	0.102**	0.244**	0.257**	0.121**	0.091**	0.231**	0.248**
Korea Republic	0.156**	0.071**	0.198**	0.203**	0.149**	0.066**	0.186**	0.196**
Latvia	0.106	0.086**	0.166**	0.177**	0.098	0.072**	0.159**	0.164**
Lithuania	0.134	0.072**	0.149**	0.165**	0.129	0.063**	0.137**	0.152**
Luxembourg	0.143**	0.085**	0.238**	0.251**	0.137**	0.080**	0.230**	0.244**
Netherlands	0.157**	0.090**	0.246**	0.264**	0.145**	0.084**	0.237**	0.255**
Norway	0.185**	0.088**	0.249**	0.261**	0.180**	0.082**	0.239**	0.249**
Poland	0.164**	0.096**	0.145**	0.154**	0.161**	0.092**	0.136**	0.143**
Portugal	0.144**	0.089**	0.223**	0.230**	0.135**	0.080**	0.219**	0.221**
Slovakia	0.158	0.092**	0.165**	0.187**	0.148	0.085**	0.155**	0.170**
Slovenia	0.131**	0.087**	0.206**	0.218**	0.125**	0.078**	0.184**	0.198**
Spain	0.139**	0.083**	0.169**	0.170**	0.132**	0.075**	0.151**	0.159**
Sweden	0.161**	0.091**	0.263**	0.281**	0.153**	0.088**	0.256**	0.267**
Switzerland	0.158**	0.095**	0.255**	0.268**	0.148**	0.090**	0.245**	0.260**
United Kingdom	0.169**	0.072**	0.223**	0.242**	0.151**	0.066**	0.208**	0.232**
United States	0.187**	0.084**	0.239**	0.245**	0.176**	0.081**	0.221**	0.238**
Panel	0.145**	0.082**	0.218**	0.223**	0.139**	0.076**	0.202**	0.214**

**significant at 5%.

**Table 12:** Long-term coefficients (Model 3 and 4).

OECD countries	Model 3				Model 4			
	SOCEX	EDEX	HEX	INCOME	SOCEX	EDEX	HEX	INCOME
Austria	0.108**	0.075**	0.198**	0.221**	0.096**	0.063**	0.184**	0.190**
Belgium	0.103**	0.064**	0.202**	0.203**	0.092**	0.051**	0.175**	0.188**
Canada	0.118**	0.058**	0.215**	0.234**	0.102**	0.045**	0.205**	0.221**
Chile	0.112**	0.073**	0.198**	0.218**	0.109**	0.059**	0.188**	0.204**
Czechia	0.128**	0.047**	0.162**	0.190**	0.117**	0.038**	0.157**	0.184**
Denmark	0.119**	0.064**	0.237**	0.237**	0.110**	0.052**	0.224**	0.229**
Estonia	0.107	0.076**	0.151**	0.157**	0.098	0.068**	0.140**	0.141**
Finland	0.116**	0.075**	0.234**	0.248**	0.105**	0.057**	0.221**	0.235**
France	0.105**	0.089**	0.237**	0.232**	0.095**	0.064**	0.229**	0.226**
Germany	0.117**	0.072**	0.231**	0.266**	0.112**	0.051**	0.213**	0.251**
Greece	0.116**	0.088**	0.218**	0.209**	0.107**	0.074**	0.185**	0.195**
Hungary	0.114**	0.065**	0.155**	0.160**	0.102**	0.050**	0.143**	0.151**
Ireland	0.165**	0.069**	0.161**	0.172**	0.147**	0.056**	0.159**	0.147**
Israel	0.188**	0.056**	0.145**	0.154**	0.172**	0.044**	0.126**	0.133**
Italy	0.101**	0.080**	0.153**	0.169**	0.089**	0.068**	0.138**	0.145**
Japan	0.107**	0.085**	0.227**	0.238**	0.090**	0.073**	0.201**	0.223**
Korea Republic	0.134**	0.057**	0.169**	0.184**	0.120**	0.043**	0.157**	0.169**
Latvia	0.088**	0.064**	0.142**	0.156**	0.071**	0.059**	0.133**	0.147**
Lithuania	0.121	0.049**	0.122**	0.141**	0.116	0.035**	0.119**	0.138**
Luxembourg	0.128**	0.075**	0.223**	0.239**	0.110**	0.057**	0.205**	0.209**
Netherlands	0.136**	0.069**	0.218**	0.240**	0.122**	0.051**	0.198**	0.224**
Norway	0.174**	0.072**	0.227**	0.232**	0.168**	0.060**	0.214**	0.201**
Poland	0.158**	0.059**	0.123**	0.130**	0.144**	0.039**	0.108**	0.117**
Portugal	0.122**	0.051**	0.198**	0.205**	0.106**	0.034**	0.181**	0.192**
Slovakia	0.137	0.070**	0.141**	0.158**	0.124	0.065**	0.130**	0.134**
Slovenia	0.118**	0.064**	0.168**	0.179**	0.101**	0.058**	0.147**	0.165**
Spain	0.121**	0.067**	0.137**	0.144**	0.115**	0.042**	0.125**	0.138**
Sweden	0.142**	0.063**	0.242**	0.255**	0.130**	0.059**	0.206**	0.223**
Switzerland	0.138**	0.077**	0.231**	0.249**	0.123**	0.071**	0.220**	0.230**
United Kingdom	0.146**	0.079**	0.196**	0.220**	0.134**	0.066**	0.174**	0.201**
United States	0.168**	0.072**	0.213**	0.228**	0.153**	0.068**	0.185**	0.217**
Panel	0.128**	0.069**	0.189**	0.204**	0.115**	0.052**	0.178**	0.182**

***, **, and * demonstrate that it is significant at 1, 5, and 10% levels, respectively.

Table 12 presents that social expenditures positively impact life expectancy at birth and 60 in nearly all OECD countries except Estonia, Lithuania, and Slovakia, but educational and health expenditures and real GDP *per capita* positively affect life expectancy at birth and 60 in all OECD members. Similarly, effect of income and health expenditures are relatively greater than impact of public social and educational expenditures. Furthermore, the long-term coefficients indicate impact of all variables on life expectancy at birth are generally higher than the impact of all variables on life expectancy at 60.

Social expenditures, including cash benefits, tax reductions, and the delivery of goods and services, can positively affect indicators of life expectancy in various ways. Firstly, cash benefits and tax reductions can increase the disposable income of individuals. This increase in personal income can support improvements in life expectancy through higher living standards, better education opportunities, improved medical care, healthier lifestyles, and reduced stress levels. Furthermore, the provision of goods and services can also contribute to life expectancy through enhanced nourishment and lifestyles. Therefore, the positive effect of public social expenditures on life expectancy across all OECD members aligns with associated theoretical views. Additionally, the results of studies by Roffia et al. (8), Dutton et al. (17), Aydan et al. (18), Cardona et al. (19), and Aydan et al. (20) on the influence of social expenditures on life expectancy in OECD countries have support our findings. Our findings also indicate that social expenditures affect not only life expectancy at birth but also healthy life expectancy at birth and 60 and life expectancy at 60 unlike these empirical studies. Furthermore, the effect of social expenditures on healthy life expectancy at birth and life expectancy at birth is usually a little higher than effect of social expenditures on healthy life expectancy at 60 and life expectancy at 60.

Education is one of the most substantial drivers of human capital, which is a key input to economic growth and development. Therefore, education can impact life expectancy through higher living standards and better health facilities. Furthermore, individuals with higher levels of education generally have greater awareness of healthy lifestyles, earn higher wages, and have better access to healthcare facilities. In conclusion, education can enhance life expectancy through these channels. This research investigates the effect of public education expenditures on life expectancy. Public education expenditures can increase life expectancy if they improve educational attainment (37). Our findings demonstrate that public educational investments enhance education and, in turn, life expectancy. However, Craigwell et al. (37) found an insignificant effect of public education spending on education. The existing empirical literature has also revealed a positive effect of various educational indicators on life expectancy (11, 22–33), and our findings largely align with the related literature and also indicate that education fosters healthy life expectancy at birth and 60 and life expectancy at 60 differently from the results of these empirical studies.

Income and health expenditures have been revealed to be the main drivers of life expectancy because health expenditures can positively affect life expectancy through improvements in health care and income can affect life expectancy through easier access to healthy life style and appropriate healthcare. Our results also reveal that income and health expenditures have relatively more effective on indicators of life expectancy. Similarly, Tarcă et al. (2), Lin et al. (38), Ali and Ahmad (39), Monsef and Mehrjardi (40), OECD (41), Aydin (42), Păunică et al. (43), Wirayuda and Chan (44), Aanegola et al. (45), Karma (46), Anwar et al. (47), Roffia et al. (8) also discovered a positive influence of income on life expectancy. On the other hand, Tarcă et al. (2), OECD (41), Aydin (42), Wirayuda and Chan (44), Aanegola et al. (45), Anwar et al. (47), Roffia et al. (8), Ahmad et al. (56), Sango-Coker and Bein (57), Gedikli et al. (58), and Khan et al. (59) also found that health expenditures fostered the life expectancy. Our study also indicates that both income and health expenditures also fostered the healthy life expectancy at birth and 60 and life expectancy at 60 unlike these empirical studies.

## 5 Conclusion, limitations, and policy recommendations

Life expectancy is one of the key indicators of a population’s health, living standards, and lifestyle. Therefore, one of the ultimate goals is to improve life expectancy in good health. In this regard, a remarkable improvement in life expectancy has been achieved globally, and the gap in life expectancy levels between countries has also partially decreased. However, a significant difference in life expectancy persists between developed and underdeveloped countries. These inequalities in life expectancy have motivated researchers to explore the economic, social, institutional, and environmental factors driving these disparities. This article investigates the long-term effect of public social and educational expenditures, health expenditures, and real GDP *per capita* on life expectancy in OECD member countries using a second-generation cointegration test.

The study includes the following limitations: First, availability of social and educational expenditures data limits our ability to perform econometric analyses for the 2005–2021 period and 32 OECD countries. Secondly, the main focus of the research is to examine the long-term effect of social, educational, and health expenditures together with real GDP *per capita* on indicators of life expectancy. Therefore, it disregards the delayed effects of the explanatory variables.

The results of the cointegration test show that public social and educational expenditures, health expenditures, and real GDP *per capita* have a positive long-term effect on indicators of life expectancy in OECD members, but real GDP *per capita* and health expenditures income are found to be more effective on indicators of life expectancy than that of public social and educational expenditures. Furthermore, the long-term coefficients also indicate that impact of all variables on healthy life expectancy at birth and life expectancy at birth are generally higher than the impact of all variables on healthy life expectancy at 60 and life expectancy at 60. Our results are largely consistent with associated theoretical views and empirical literature, but they also indicate that public social and educational expenditures, health expenditures, and real GDP *per capita* impact not only life expectancy but also healthy life expectancy.

Based on our empirical findings, social, educational, and health expenditures, and real GDP *per capita* are significant drivers of life expectancy and healthy life expectancy. Therefore, efficiently utilization of public social and educational expenditures and health expenditures can be used to improve life expectancy. Furthermore, improvements in social, educational, and health expenditures can also foster life expectancy through economic growth and development. Future studies could explore the decomposition of social, educational, and health expenditures’ effects on indicators of life expectancy.

## Data Availability

The original contributions presented in the study are included in the article/supplementary material, further inquiries can be directed to the corresponding author.
